# Estimating genetic parameters of egg production in three local Tanzanian chicken ecotypes

**DOI:** 10.1016/j.psj.2026.106751

**Published:** 2026-03-13

**Authors:** T. Magwaba, J.C.M. Dekkers, S.J. Lamont, A. Wolc, E. Mollel, J.R Mushi, M. Walugembe, E.N Amuzu-Aweh, G.H. Chiwanga, N. Chouicha, P.L. Msoffe, T. Kelly, R.A. Gallardo, A.P. Muhairwa, H. Zhou

**Affiliations:** a Iowa State University, Ames, IA 50011, USA; b Sokoine University, P.O. Box 3000, Chuo Kikuu, Morogoro, Tanzania; c University of Ghana, P.O. Box L.G. 25, Legon, Accra, Ghana; d Department of Animal Science, University of California, Davis One Shields Avenue, Davis, CA 95616, USA; e Hy-Line International, Dallas Center, IA 50063, USA

**Keywords:** Indigenous chicken, Egg production, Heritability, Genetic parameter, Clutch

## Abstract

Indigenous chickens (**ICs**) play a crucial role in ensuring food security, generating income, and supporting livelihoods in many developing countries. Despite their importance, ICs typically exhibit low productivity, particularly in egg production. Understanding their genetic potential is essential for developing sustainable breeding strategies. This study estimated genetic parameters for egg production and egg weight traits in three Tanzanian IC ecotypes: Ching'wekwe (*n* = 171), Kuchi (*n* = 220), and Morogoro Medium (*n* = 406). Birds from 13 hatches were raised under improved conditions, and egg production data were collected across six consecutive 90-day periods (**EP**1 to EP6), beginning at the age of first egg (**AFE**; mean = 164 ± 15.5 days). Laying status (**LAY**) was defined per period, with LAY = 1 if >3 eggs were laid and LAY = 0 otherwise. Additional egg production traits included average clutch length (**ACL**), number of eggs per clutch (**EPC**), and inter-clutch pause (**ICP**). The average annual egg production was 52.1 eggs per hen, and mean egg weight (**EW**) was 43.1 ± 5.5 g, peaking in EP5. Ecotype significantly affected most EW traits, with Kuchi hens laying the heaviest eggs and showing shorter clutch lengths and pauses (*p* < 0.05). Heritability estimates ranged from 0.1 to 0.6 for EP, from 0.1 to 0.4 for EW, from 0.1 to 0.2 for clutch traits, and from 0.1 to 0.3 for LAY. Genetic correlations between egg production in different periods were mainly positive (average: 0.38), and those among EW traits in various periods were strong and positive (average: 0.80). Clutch traits showed mixed or weak genetic correlations. Despite low productivity, the observed moderate heritabilities and positive genetic correlations between periods suggest scope for selection-based improvement. However, some unfavorable genetic correlations and age-related variability highlight the need for flexible, multi-trait selection strategies to balance production traits over time.

## Introduction

Indigenous village chickens (**ICs**) are typically raised in harsh environments, providing low-cost meat and egg-based protein for low-income households in developing countries (Desta, 2021b). The ability of ICs to perform under such environments significantly sustains food security, nutrition, and livelihoods for rural communities. ICs also have region-specific cultural values that are fundamental in the communities in which they are raised (Desta, 2021a; Siripurapu and Das, 2018). The ICs have also been considered potential biological pest and weed-controlling agents in low-resource mixed crop-animal production systems, reducing the financial requirements associated with chemical pest control methods (Sean et al., 1996). While these positive aspects of ICs have led to their widespread adoption in resource-challenged parts of the world, their low egg and meat production performance has raised sustainability concerns (Kpomasse et al., 2023).

Tanzania has a low annual egg consumption of 1.5 kg per capita (Ministry of Livestock and Fisheries, 2019), which is significantly below the recommended 9.35 kg per year consumption by the World Health Organization (Giannetto et al., 2016). Without alternative interventions, anticipated population increases, land fragmentation (Bizimana, 2009), and climate change are expected to reduce further the quantity of products realized from ICs (Gitz et al., 2016). The need to increase IC productivity is also driven by the recent surge in demand for animal-based protein and by the preference for white over red meat due to health concerns (Henchion et al., 2017).

While the low production performance of ICs is beyond dispute, this assertion often ignores the production environment in which ICs are raised. For instance, most ICs are raised in poor conditions or without housing infrastructure, eliminating essential production-promoting factors such as lighting control, temperature, and biosecurity. Also overlooked is the brooding time IC hens invest in providing hatching conditions for their eggs. An objective comparison of the production performance of ICs and commercial chickens must account for these differences.

Evaluating egg production performance in chickens requires phenotyping multiple traits. Phenotyping must be accurate, requiring large datasets for practical genetic analysis and improvement. This is essential for estimating heritabilities and genetic correlations, as well as for implementing targeted breeding strategies to achieve optimal performance. Age at first egg (AFE) is one of the traits that influences overall production efficiency and is known to have a negative genetic correlation with the number of eggs laid from a given age across all chicken production systems (Yang et al., 2023; Hicks, 1958; Firozjah et al., 2015). Egg weight is correlated with egg size and the egg's visual appearance, which, in turn, affect production efficiency, market value, consumer perception, hatchability, and chick survival (Williams, 1994).

Recording the number of eggs laid over a given period forms the basis for quantifying egg production in chickens. While this practice is common in commercial settings, it is less common in extensive and semi-extensive systems (Muchadeyi et al., 2009), making it challenging to improve such traits in ICs due to a lack of records. The most intuitive definition of egg production is the number of eggs laid from the first egg to the end of the laying period. The recording of egg numbers must account for differential gene expression across production stages (Liljedahl et al., 1999), resulting in less than one genetic correlation estimate between egg production records collected at different stages of the production cycle (Wolc et al., 2007). Part-period egg production recording and analysis are widely used to account for genetic differences in the control of egg production over time (Wolc et al., 2007; Dana et al., 2011).

Unlike in ICs, commercial layer breeding programs have applied selection pressure on the number of eggs laid for many years, resulting in egg production rates in breeding populations becoming more uniform, given the physiological upper limit of 1 egg that a hen can lay per day, which has significantly reduced the genetic variance for egg production (Wolc et al., 2007). Alternative traits, such as clutch length, number of eggs per clutch, and interclutch pause, are more heritable than the total number of eggs and exhibit stronger associations with specific genomic regions (Wolc et al., 2019). While clutch traits are also considered essential for egg production of ICs (Yakubu et al., 2008; Yang et al., 2023), they serve different purposes in IC production systems than in commercial systems, as they define laying cycles and brooding periods in ICs. The brooding period is crucial for ICs, as it ensures a conducive temperature and humidity for eggs to hatch and for the chicks shortly after hatching. The presence of brooding behavior in IC production systems and its absence in commercial egg production systems partly explains the difference in the number of eggs produced between ICs and commercial hens.

The full egg-production potential of various ICs has not been evaluated due to the limited phenotypic data required for such studies. We raised three Tanzanian ecotypes under improved conditions to collect the data required to address this issue. This enabled us to evaluate their current production status and estimate genetic parameters for production potential, thereby establishing a foundation for improving egg production through genetic selection. Thus, this study aimed to estimate genetic parameters for egg production of three IC ecotypes from Tanzania under improved management conditions that enabled phenotyping of hens, providing records typically unavailable from small-scale farmers and households that raise these chickens. Knowledge of genetic parameters and variance components is crucial for understanding the factors that contribute to phenotypes, understanding trait inheritance, and developing effective breeding and selection programs. In this study, egg production traits evaluated included part-period egg number (EP), egg weight (EW), laying status (LAY), and clutch-related traits (ACL, EPC, ICP), which are commonly used traits for genetic evaluation of laying performance.

## Materials and methods

Approval for all animal procedures was granted by the University of California-Davis Institutional Animal Care and Use Committee (protocol #17853). Egg production data were collected from July 2019 to December 2021 on 1136 hens that were hatched from January 2019 to August 2020 in 13 hatches. The hens were offspring of breeders collected from various regions of Tanzania, representing three Tanzanian chicken ecotypes, Ching'wekwe, Kuchi, and Morogoro-medium, as described in Mushi et al. (2020). Upon hatching, chicks were weighed, wing-tagged, and housed in brooding facilities until they were 3 weeks of age. Thereafter, they were transferred to aviaries with artificial lighting, where they were raised under high biosecurity conditions, with equipment disinfected and restricted access. At 17 weeks of age, female birds were transferred to individual cages and remained there for the entire laying period, without forced molting. Disease control measures were implemented in accordance with the protocols of the Tanzanian government's veterinary department. Newcastle Disease vaccine was administered at 3 and 21 days of age, and then every three months thereafter. Gumboro vaccinations were administered at 11 and 18 days, and Fowlpox vaccination at 8 weeks. Water and commercial feed were provided ad libitum. Table 1 presents the feed composition for chicks (0-8 weeks), growers (8-20 weeks), and layers (over 20 weeks of age). Incandescent bulbs provided artificial lighting when natural light was unavailable. Beak trimming was performed at 7 days of age using an electric hot blade and then whenever necessary to avoid feather pecking and cannibalism.

**Table 1:** Composition of chicken feed at different stages of production.

	Inclusion rate (%)		
Ingredient	Chicks (0-8 weeks)	Growers (9-19 weeks)	Layers (≥ 20 weeks)
Maize	46	14.3	45
Maize/Wheat Bran	13	24.3	19
Rice/Wheat Polish	10	32.9	2
Fish Meal	2.6	4.9	10
Sunflower Cake	25	15.7	16
Limestone	2.5	2.9	3
Lysine	-	-	0.25
Bone Meal	-	1.0	2.5
Dicalcium phosphate	-	-	1.5
Methionine	-	-	0.25
Salt	0.5	0.5	0.5
Premix	0.4	3.5	-

**Fig. 1:**
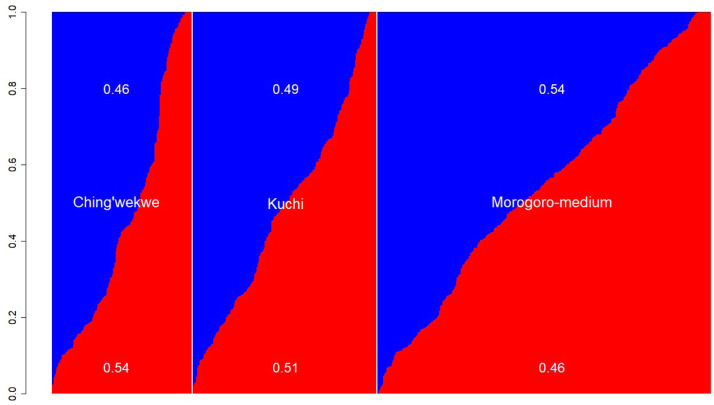
Admixture graph for hens sorted by ancestral proportion within ecotype.

Blood samples from all birds were submitted for genotyping using targeted genotyping by sequencing at 5 K SNPs, as described by Kanlisi et al. (2022), and imputed to the Affymetrix chicken 600 K SNP chip using FImpute (Sargolzaei et al., 2014). Pairs of hens (*n* = 13) with a genomic relationship above 0.8 were removed from the data to exclude individuals with potential sample mix-ups or duplicate sampling (Douglas et al., 2002; Pompanon et al., 2005). Hens with no genotype data (*n* = 8) and hens with incomplete records (*n* = 308), such as missing hatch date or AFE, were removed. After quality control, 797 hens remained for analysis, comprising 406 Morogoro, 171 Ching'wekwe, and 220 Kuchi hens. Admixture analyses of genotypes using the LEA software (Frichot and François, 2015) grouped these three ecotypes into two distant ancestral populations, as shown in Fig. 1.

All eggs laid from July 2019 to December 2021 were collected daily and weighed using a digital scale. Data were processed in R version 4.2.2 (R Core Team, 2022) to examine descriptive statistics. The egg production (**EP**) data were summarized as the total number of eggs laid over consecutive 90-day periods, starting from the hen's age at first egg (AFE), and designated EP1 through EP10. Production data for periods 7 to 10 were excluded from the analyses due to insufficient data. EP records for hens that died before completing the 90 days of any specific period were set to missing. Distributions of the EP phenotypes deviated from normality, with many records having 3 or fewer eggs per 90-day period. To address this skewness, records with three or fewer eggs were set to missing, and a binary trait, **LAY**, was created for each EP to distinguish between low layers and productive layers. By period, birds with 0 to 3 eggs were categorized as LAY=0, and those with more than three eggs were classified as LAY=1. The numbers of records with LAY=0 for each period are shown in Table 2**.** For egg weight, eggs weighing less than 10 g and more than 80 g were considered outliers (*n* = 15) and were set to missing. Egg weights were averaged for every 90 days (EW1 to EW6), regardless of whether LAY was 0 or 1.

**Table 2:** Descriptive statistics for the number of eggs laid in consecutive 90-day periods (1 to 6), starting from age at first egg by LAY^1^ category.

	LAY = 0				LAY = 1						Overall	
Period	n^2^	%^3^	Mean	SD^4^	n	%	Min^5^	Mean	Max^6^	SD	Mean	SD
1	137	17.2	1.5	0.7	660	82.2	4	24.2	61	11.1	20.25	13.21
2	110	13.8	0.6	0.9	413	51.8	4	15.4	53	8.8	9.04	9.90
3	79	9.9	0.6	1.0	317	39.8	4	15.7	40	7.7	9.31	9.48
4	75	9.4	0.7	1.0	309	38.8	4	19.3	48	11.0	12.16	12.51
5	47	5.9	0.8	1.0	287	36.0	4	20.7	58	10.9	15.42	12.83
6	33	4.1	0.9	1.1	263	33.0	4	14.9	47	7.9	11.92	9.06
**Average**		**0.9**	**-**			**-**	**18.4**	**-**		**13.32**	**-**	

1 LAY=1 - hens which laid >3 eggs, LAY=0 – hens which laid ≤3 eggs.

2 n – the number of hens in that period by LAY category.

3 % – number of hens as a percentage of the total number (*n* = 797) of layers in the experiment by LAY category.

4 SD – standard deviation.

5 Min – minimum number of eggs laid for that period.

6 Max- maximum number of eggs laid,.

**Fig. 2:**
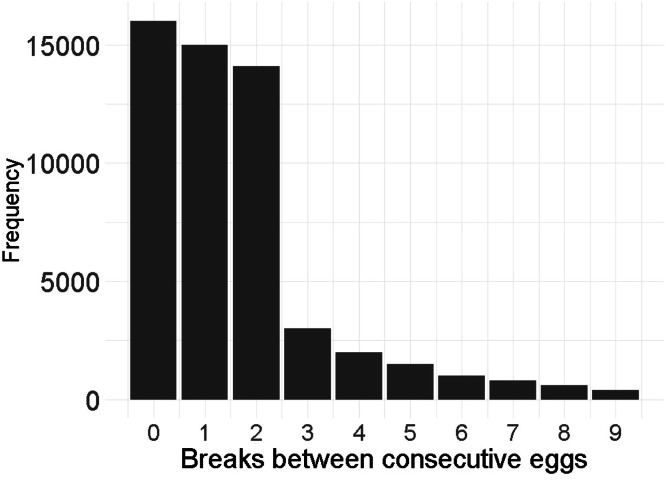
Distribution of the number of days between successive eggs.

We also analyzed age at first egg (AFE) and average clutch traits across the entire recording period, including average clutch length (**ACL**), the average number of eggs per clutch (**EPC**), and the average inter-clutch pause (**ICP**). The distribution of the number of days between subsequent eggs was examined to define clutch traits, and a gap of 1 or 2 days was deemed an appropriate time lapse between successive eggs to identify clutches (Fig. 2). A clutch was, therefore, defined as a continuous egg-laying period with no more than a two-day gap between successive eggs. Outliers for average ICP (*n* = 3) were identified as values exceeding 1.5 times the interquartile range (IQR) and removed.

### Statistical analyses

Univariate and bivariate mixed linear models were used to estimate genetic parameters for all traits, utilizing the ASReml software version 4.2 (Butler et al., 2023) and applying the following mixed linear model as described in Henderson and Quaas (1976) as cited in Magwaba et al. (2022);

y=Xb+Zu+e

where **y** is the vector of phenotypes; **X** and **Z** are incidence matrices for fixed and random effects, respectively; **b** is a vector for fixed effects; **u** is a vector of random genetic effects with covariance structure **G**σ^2^**_g_**, where **G** is the genomic relationship matrix based on method 1 of VanRaden (2008) and σ^2^_g_ is the genetic variance; and **e** is a vector of residuals with variance **I**σ^2^_e,_ where **I** is the identity matrix and $\sigma_{e}^{2}$ is the variance of the residuals. Residuals for analyses of average EW within a period as a phenotype were weighted by the number of eggs in the corresponding 90-day period. Fixed effects in the univariate model included hatch date and ecotype as class variables, and AFE and ancestral proportion as covariates.

Heritability and correlation estimates for the binary trait LAY were converted to the liability scale using the following transformation formula by Lee et al. (2011)

$$h_{l}^{2}=h_{o}^{2}\frac{K\left(1-K\right)}{z^{2}}$$

where $h_{l}^{2}$ is the heritability on the liability scale, $h_{o}^{2}$ is the heritability of the observed scale, z is the height of the normal curve at the threshold, and *K* is the proportion of hens with LAY =1.

For AFE, values below 110 (*n* = 11) and above 190 (*n* = 290) were treated as outliers and set to missing when AFE was analyzed as a trait; however, they were included when AFE was fitted as a covariate for other traits. The distribution of AFE before and after removing outliers is shown in Table 3.

**Table 3:** Descriptive statistics for age at first egg, average clutch length, inter-clutch pause, and eggs per clutch.

Trait	Number of records	Minimum	Mean	Maximum	SD^1^
Age at first egg (with outliers)	797	79.0	184.5	539.0	47.6
Age at first egg (no outliers)	516	110.0	164.2	190.0	15.5
Average clutch length (days)	797	12.0	5.3	164.0	2.6
Eggs per clutch (eggs)	797	11.4	3.6	140.0	1.8
Inter-clutch pause (days)	786	3.9	32.5	138.7	2.9

1 SD – standard deviation.

Bivariate analyses of the above models were used to estimate phenotypic and genetic correlations between traits. For bivariate analyses involving AFE, the other trait was pre-adjusted for AFE to avoid numerical issues that can arise when a variable is included both as a trait and a covariate in multivariate analyses. The pre-adjustment was based on the univariate model estimate of the regression coefficient of the trait on AFE.

Most bivariate models that included both trait means and individual trait weights failed to converge. To address this, egg weight data were analyzed as individual egg weights measured repeatedly over time. Hen was included as a permanent environmental effect. In the bivariate analyses based on this framework, residual covariances between repeated records from the same hen were set to zero. For traits in which the permanent environmental variance converged to zero in the univariate analyses, bivariate models were initially run with the covariance between permanent environmental effects included. Models that failed to converge under these conditions were subsequently refitted with the covariance between permanent environmental effects set to zero. For traits that did not converge, the phenotypic variance was estimated by excluding the random genetic effect from the model.

## Results and discussion

### Descriptive statistics

Summary statistics for the edited dataset are presented in Table 2, Table 3, Table 4. The number of hens in lay decreased from 797 in period 1 to 296 in period 6, representing 62.9% mortality (Table 4). Pre-lay mortality records were not available. Kumar et al. (2016) reported a higher mortality rate (69.4%) in free-range systems in India from hatch to 72 weeks of age. In their study, some deaths were attributed to predation, which was absent in our study. In Kenya ICs, 60% mortality was reported across different age groups over 13 weeks (Otiang et al., 2020). The high mortality rate in our study is a concern, especially given the improved conditions under which the hens were raised. Mortality is expected to be higher in free-range systems because of relaxed biosecurity measures, limited veterinary services, and the prevalence of predation (Desta, 2021a; Mujyambere et al., 2010). The high mortality losses and the absence of proper diagnostics highlight the need to improve laboratory capacity and expertise to enhance animal health, biosecurity, and veterinary services.

**Table 4:** Descriptive statistics for egg weight (g) by 90-day period (1 to 6) from age at first egg.

Period	Number of hens	Minimum	Mean	Maximum	Standard deviation
1	797	27.0	42.8	62.9	5.0
2	523	27.0	43.6	66.0	5.2
3	396	29.2	43.9	64.3	5.6
4	384	18.5	40.6	57.1	6.1
5	334	30.0	44.7	63.7	5.3
6	296	33.0	44.1	61.7	4.9
Average			43.1		5.5

Based on Table 2, the average number of eggs laid per hen declined by 15.5% from period 1 to period 2, then increased to approximately 20 eggs per hen in period 5, before declining to below 15 eggs per hen in period 6. Summary statistics for LAY showed a decrease in the number of hens for LAY=1 and LAY=0 as time in lay progressed (Table 2). The proportions of hens within LAY=0 from period 1 to period 6 were, respectively, 17, 21, 20, 20, 14, and 11% of the total number of hens. In this category, most hens (*n* = 90) did not lay any eggs during that period, while some laid two (*n* = 23) or three (*n* = 18) eggs. The mean number of eggs laid per 90-day period per hen was 0.9 for hens in the category LAY=0 (periods where a bird laid >3 eggs) and 18.4 for periods with LAY=1 (Table 2). The latter is expected to be even lower under free-range conditions, a typical environment for ICs, due to limited and low-quality feed and nutrient supply, disease, and parasite infestation, among other factors. The average rate of lay was 41.5 eggs in the first year after the first egg, which is approximately 10% lower than the 45.5 eggs reported by Guni et al. (2013) in their free-range study across three districts in Tanzania. Still, it falls within the 41-55% range reported by CSIRO (2015) for Tanzanian local chickens. Further studies are required to determine if confinement negatively impacts the egg-laying performance of indigenous chickens, as it is not their natural environment. The average performance in our study is substantially lower than the average egg production rates in commercial systems. For example, it is 18.7% of the average number of eggs per hen per year in the USA (USDA, 2022). This confirms reports that indigenous chickens exhibit poor egg production performance (Desta, 2021b; Kpomasse et al., 2023; Manyelo et al., 2020; Muchadeyi et al., 2009; Safalaoh, 1997). However, the difference in production between commercial chickens and ICs must be considered in light of resource input, production, and biological aspects. For example, IC hens brood over their eggs for incubation and have been naturally selected for brooding, a practice absent in commercial layers. Although hens did not brood over their eggs in this study, the quality of the environment, the housing (less wind and temperature [roof), lighting (light sources and duration), and biosecurity management standards were poorer than in most commercial farms.

In the raw data, the minimum AFE was 79 days, the average was 184.6 days, and the maximum was 539 days (Table 3). For analysis of AFE as a trait, we used only phenotypes ranging from 114 to 190 days, with an average of 164.2±15.5 days (mean±SD). This range was deemed optimal, as including AFE records with values outside it substantially reduced the heritability estimate for AFE. A similar average of 162.6 days was reported in a survey of various ICs in extensive production systems across two counties in Kenya (Atela et al., 2016). A slightly lower average AFE of 155 days was reported for Indian ecotypes in the free-range systems survey by Kumar et al. (2016). Our finding corroborates earlier reports that ICs lay later than commercial breeds, which start laying at around 133 days of age (Lohman Breeders, 2021). A later AFE increases production costs and the time it takes to generate returns on investment.

The average egg weight across the six periods was 43.1 ± 5.5 g (Table 4). There was a general increase in average egg weight from 42.8 g in period 1 to 44.1 g in period 6, except for a drop in average EW by 3.3 g in period 4. The average EW in this study is comparable to the 45.1 ± 8.5 g reported by Olwande et al. (2010) for Kenyan ecotypes under an extensive production system and to the 43.3 ± 6.2 g reported in India by Kumar et al. (2016). The average EW in period 6 (44.1 g) was approximately 68% of the mean EW (64.8 g) of Hy-Line Browns at 72 weeks of age (Hy-Line, 2024). Combining fewer and smaller eggs laid by the ecotypes under study results in substantially fewer kilograms of protein for human consumption per year. This lower production level of ICs can be attributed to their reproductive physiology, which prioritizes survival traits over reproduction, and to their lack of selective breeding for egg production traits.

The average clutch traits exhibited considerable variation among hens (Table 3). The average EPC was 3.6, the average CL 5.3 days, and the average ICP 32.5 days. Of all hens, 10.9% and 5.2% had ICP exceeding 60 and 90 days, respectively. A longer ICP is crucial for ICs, as hens naturally brood over their eggs during this period. Brooding is replaced by hatcheries in commercial production, leading to a gradual decline in brooding-related behaviors. Based on resource allocation theory, Beilharz et al. (1993) suggested that commercial hens were bred to prioritize egg laying at the expense of other traits observed in ICs, such as brooding. This leads to reduced allocation of energy resources towards brooding.

The fluid definition of a clutch presents a challenge for a fair comparison between studies. In this study, the average EPC was 3.88 eggs, which is far lower than the 10-20 eggs reported by Leta and Bekana (2010) for Ethiopian ICs. The differences may be due to the definition of a clutch, which was not provided in the studies referred to here. In our study, a clutch was defined based on the observed laying patterns in our data (Fig. 1), with a maximum 2-day gap permitted between subsequent eggs. This definition differs from that of Nalbandov (1976) and Roy et al. (2014) for commercial populations, which limit the maximum time between consecutive eggs within a clutch to 1 day. However, environmental conditions are maintained relatively constant under commercial production, including feed and temperature. Such strict environmental control is absent for ICs in this study, as they experienced high environmental variability due to the housing design and inability to maintain a constant environment, exacerbated by frequent power cuts, inconsistent input supply (feed, drugs, lighting), and non-commercial house design. Given the genetic differences due to selection history, which influences the laying pattern and hence the observed frequency distribution of days between eggs (Fig. 2), we adopted a relevant definition of a clutch for this study that differed from most literature.

Analysis of ancestral proportions derived using the LEA software as the proportion of alleles an individual carries that trace back to the blue ancestral population in Fig. 1, revealed that the average proportions were 0.46, 0.49, and 0.54 for Ching'wekwe, Kuchi, and Morogoro-medium, respectively, and were significantly different between the ecotypes (*p* < 0.0013). There was no difference between Kuchi and Ching'wekwe, whereas the ancestral proportion for Morogoro-medium was higher than that of Ching'wekwe (*p*≤ 0.001) and Kuchi (*p*≤0.05).

### Estimates of fixed effects

Hatch date had a significant effect on most traits analyzed (*p* < 0.015), except for EW in periods 4 to 6 (Table 5), suggesting that the time of year when chicks hatch affects early-laying performance. Hatch date is inherently confounded with various environmental and management factors, reflecting the combined effects of all conditions influencing a contemporary group relative to others. Phenotypes expressed later in life are linked to conditions individuals are exposed to early in life; for example, feed restrictions and adverse temperatures influence mature body weight (Shinder et al., 2002). Tzschentke and Basta (2002) demonstrated that exposure to thermal stress during the perinatal period (soon after hatching) can trigger epigenetic adaptations that alter the thermoregulatory threshold of the preoptic anterior hypothalamus, a key brain center controlling body temperature. Additionally, synchronization between production status and the time of year is crucial, as circannual rhythms strongly govern the reproductive and laying cycles of ICs under non-controlled housing conditions. Hens tend to lay more eggs during periods of increasing daylight and warmer temperatures (Er et al., 2007; Oso et al., 2022). These environmental factors affect egg production, highlighting the influence of seasonal variations on laying behavior in such systems.

**Table 5:** Estimates and standard errors (SE) of the effects of age at first egg, ancestral proportion, and ecotypes on egg production traits by 90-day period (1 to 6) from age at first egg. The estimates and standard errors for LAY1 to LAY6 are expressed as percentages.

Trait	Age at first egg		Ancestral proportion		Ecotype (deviated from Morogoro)				Estimates		
					Kuchi		Ching'wekwe				
	Effect	SE	Effect	SE	Effect	SE	Effect	SE	Kuchi	Ching	Moro
EP1	−0.033***	0.01	6.02	1.92	0.76	0.79	0.58	0.90	22.64	22.28	22.18
EP2	−0.01	0.01	3.74	2.44	−0.60	0.95	−0.54	1.06	14.21	12.96	13.80
EP3	−0.0001	0.01	2.26	2.24	0.10	1.09	−1.59	1.05	10.79	9.03	10.80
EP4	−0.01	0.02	4.76	3.82	0.99	1.45	−2.31	1.41	8.72	5.28	7.97
EP5	−0.044**	0.01	5.31	4.18	0.14	1.51	1.72	1.46	22.11	23.53	22.24
EP6	−0.033**	0.01	6.11	3.60	0.15	1.20	−0.38	1.14	15.55	14.84	15.71
LAY1	−006***	0.05	0.01	0.10	1.4**	1.50	−10.50**	3.50	0.71	0.59	0.70
LAY2	−0.05**	0.06	0.07	0.14	3.91**	3.19	−2.56**	2.28	0.43	0.36	0.39
LAY3	−0.02**	0.06	0.04	0.14	−0.02	0.04	0.01	0.04	0.27	0.30	0.29
LAY4	−0.07***	0.05	0.05	0.11	−0.04	0.03	−0.01	0.04	0.28	0.30	0.32
LAY5	−0.13**	0.05	0.04	0.10	−0.04	0.03	−0.08	0.03	0.27	0.23	0.31
LAY6	−0.13**	0.05	0.06	0.07	−0.02	0.03	−0.04	0.03	0.24	0.29	0.26
EW1	0.001	0.004	4.47	2,43	0.99***	0.44	−2.71**	0.46	44.61	40.78	43.84
EW2	−0.002	0.01	6.0***	2.1	−0.49	0.50	−1.17	0.55	45.05	44.19	45.84
EW3	0.01	0.01	7.5***	2.2	0.84***	0.55	−1.61***	0.54	46.08	43.41	45.61
EW4	0.01	0.01	8.88***	1.85	0.16**	0.56	−1.67**	0.55	37.95	35.85	38.24
EW5	0.01	0.01	8.51***	2.18	−0.85*	0.60	−1.60*	0.58	42.57	41.56	43.86
EW6	0.01	0.0	6.18***	1.75	−1.57***	0.60	−1.54***	0.58	41.22	42.06	43.10
ACL	−0.061***	0.018	0.53	0.02	0.65**	0.20	0.28**	0.13	5.64	4.46	4.47
EPC	−0.037***	0.013	0.45	0.12	0.41*	0.14	0.16*	0.10	3.50	3.17	3.12
ICL	−0.02	0.01	6.35	2.41	−2.9	2.3	6.46	2.47	53.67	63.33	55.99
AFE	−0.15	0.02	5.22	3.82	−1.4	2.1	7.23	5.32	168.33	166.32	168.09

EP1 – the first 90-day laying period from age at first egg, LAY1 – LAY for period1, EW1 (g) – Average egg weight for period1 in grams, ACL – average clutch length in days, EPC – egg per clutch, ICP – inter clutch pause in days, AFE – age at first egg in days, SE – standard error.

Probability values: *P≤0.05, **P≤0.01 and ***P≤0.001.

Ancestral proportions significantly affected EW in periods 2-6, while ecotype affected LAY in periods 1 and 2, EW in all periods except 2, ACL, and EPC (Table 5). In period 1, Kuchi laid the most eggs (22.2), while Morogoro-medium laid the heaviest eggs on average (43.4 g), and Kuchi had the longest ACL. Ching'wekwe had the largest number of eggs per clutch. Ancestral proportion and ecotype are confounded and, therefore, their combined effects will be discussed. Ancestral differences arise from different alleles inherited from the two ancestral populations identified through admixture analysis (Fig. 1), while ecotypes reflect the combined influence of genetics and environment on phenotypes. The inclusion of both ancestral proportions and ecotypes in the analyses represents a beneficial redundancy, in which one factor compensates for effects missed by the other.

Within species, differences in production performance among individuals with different genetic backgrounds and habitats are well-documented (Biscarini et al., 2010; Muchadeyi et al., 2009). Ecotype differences in egg weight, AFE, and egg number were also reported by Lwelamira et al. (2008) for Kuchi and Morogoro-medium, with Kuchi laying more eggs earlier under improved management. These observations were confirmed in the current study.

AFE significantly and negatively affected EP in periods 1, 5, and 6, LAY in all periods, ACP, and EPC, but did not affect EW (Table 5). Hens raised in conducive environments with moderate temperatures and ample light exhibit faster growth and development, resulting in a lower AFE than those raised in colder, low-light conditions (Cui et al., 2019). The reduced time to laying is facilitated by activation of the hypothalamus-pituitary-gonad axis, which promotes the release of reproductive hormones and accelerates sexual maturity (Kuohung and Kaiser, 2006). Our results imply that early-laying hens tend to lay more eggs. Published studies have suggested that hens that lay eggs earlier often exhibit greater physiological readiness, driven by reproductive hormones such as gonadotropin-releasing hormone (Kuohung and Kaiser, 2006).

### Estimates of heritabilities and repeatabilities

**Fig. 3:**
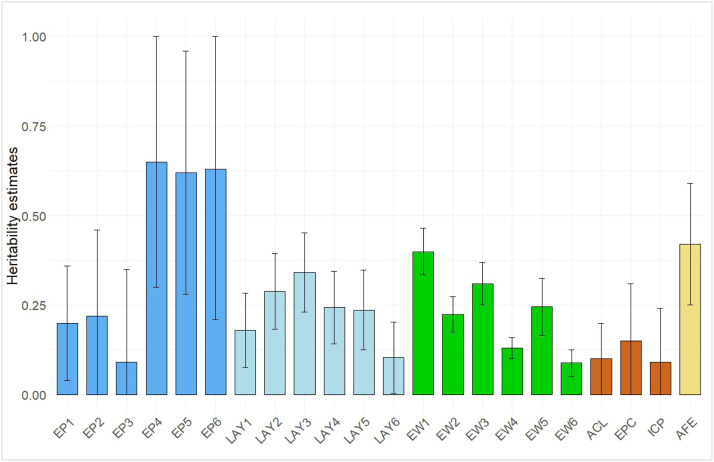
Estimates and standard error bars of heritability for egg production traits. EP – egg production for period 1-6 EW – egg weight for periods 1-6 AC – average clutch length EPC – eggs per clutch ICP – inter clutch pause AFE – age at first egg.

Heritability estimates for early EP in periods 1 to 3 were generally low, ranging from 0.09 to 0.22, and increased in the later periods, EP4 to EP6 (0.62 to 0.65) (Fig. 3). Specifically, EP1 had the lowest heritability estimate, while EP6 exhibited the highest. Progressive partial production records have been utilized in other studies. (1999) used partial 5-day periods in egg production studies with commercial layers and concluded that egg production heritability increased with age. Singh et al. (2018) observed increasing heritability estimates for egg production in Indian Uttara ICs from 0.14±0.05 (± standard error) at 40 weeks to 0.24±0.20 at 54 weeks. This generally places our estimates within published ranges and supports the observed trend of increasing heritability with age.

Heritability estimates for LAY ranged from 0.10 to 0.34 on the observed scale (Table 6). Although recorded on a binary scale, the trait LAY is influenced by an underlying continuous latent scale (liability) that integrates genetic and environmental factors determining a hen's category (Falconer and Mackay, 1996; Lee et al., 2011). Heritability estimates on the liability scale ranged from 0.17 to 0.55, indicating that genetic factors contribute to the underlying propensity to lay eggs in hens, even though this may not be immediately apparent from the observed binary outcome. Analyzing a binary variable increases environmental variance, reducing genetic variance (Falconer and Mackay, 1996; Speed and Evans, 2024). The genetic variance obtained when a binary trait is analyzed as continuous depends on the trait's prevalence, and this bias can be corrected by converting the observed trait to a liability scale (Dempster and Lerner, 1950). Selection for traits like LAY should ideally utilize information about the underlying liability, i.e., through genetic markers and/or pedigree information, rather than relying solely on the observed binary outcome.

**Table 6:** Estimates of heritabilities and genetic correlations for LAY traits on the observed (o) and liability (l) scales.

			Heritabilities		Genetic correlations	
Period	K	Z	h^2^_o_	h^2^_l_	t_o_	t_l_
1	0.83	0.25	0.18	0.40	0.12	0.27
2	0.52	0.40	0.29	0.46	0.03	0.05
3	0.40	0.39	0.34	0.55	0.13	0.21
4	0.39	0.38	0.24	0.39	−0.17	−0.27
5	0.36	0.37	0.24	0.40	0.39	0.64
6	0.33	0.36	0.10	0.17	−0.24	−0.40

K is the prevalence of LAY=1, z - the height of the normal curve at the threshold, $h_{o}^{2}$ is the heritability of the observed scale, $h_{l}^{2}$ is the heritability on the liability scale, $t_{o}$ the correlation on the observed scale, $t_{l}$ the correlation on the liability scale.

The clutch traits ACL, EPC, and ICP had heritability estimates of 0.16±0.09, 0.13±0.08, and 0.18±0.10, respectively (Fig. 3). Yang et al. (2023) reported estimates of 0.38 ± 0.05 for ACL and 0.05 ± 0.03 for ICP in indigenous Beijing-You chickens. Several factors may have contributed to these differences. Firstly, the definition of a clutch differs across studies; Yang et al. (2023) allowed no more than 1 day between successive eggs in a clutch, whereas this study allowed up to 2 days. Additionally, factors such as the purpose and breeds of the hens, the rearing environment, and selection histories may affect heritabilities. Moreover, the analyses were conducted using datasets of varying sizes, with typically large standard errors, particularly in IC studies, due to limited data.

The heritability estimate for AFE was relatively high at 0.42±0.17 (Fig. 3). This estimate was affected by the inclusion of AFE values exceeding 190 days. The heritability for AFE in this study was significantly higher than the estimate of 0.06±0.15 reported by Dana et al. (2011) for Horo local chickens in Ethiopia under improved management. A higher estimate of 0.62±0.04 was reported for the Beijing-You indigenous chickens by Yang et al. (2023). Our study's high heritability estimate for AFE indicates sufficient genetic variability to select for ICs with early egg laying.

Among the EW traits, EW1 had the highest heritability of 0.40, while EW6 showed the lowest estimate of 0.09 (Fig. 3). When egg weights were analyzed as individual egg weights using a repeatability model, the estimates ranged from 0.13 (EW1) to 0.51 (EW4), and repeatability estimates ranged from 0.44 to 0.64 (Table 7). The range of heritability estimates for EW obtained in this study is comparable to previously published results, including an estimate of 0.27±0.08 for Brazilian commercial laying chickens reported by Bogdanski et al. (2023) and estimates ranging from 0.34 to 0.40 for EW between 28 and 32 weeks of age of Iranian native chickens by Firozjah et al. (2015). Unlike egg production, which showed an increasing trend over time, heritability estimates for egg weight generally declined from period 1 to 6 (Fig. 3). Other researchers have confirmed a general decrease in the heritability of egg weight with age (Chandan et al., 2019). Moderate heritability estimates for EW across four of the six periods in our study suggest the possibility of improving these traits through selection. Repeatability estimates for EW (Table 7) are much higher than those reported for Bovan Nera Black chickens in Nigeria by John-Jaja et al. (2016) (0.19±0.03 to 0.21±0.05), with repeatability estimates increasing as time in lay progresses. These differences could be attributed to differences in genetic background, management practices, or environmental conditions between the two studies. High repeatability estimates in our study suggest that genetic and general environmental factors are consistent across periods.

**Table 7:** Estimates of repeatability and their standard errors for individual egg weights by 90-day period from the start of lay based on a repeatability model.

Period	Repeatability	Standard error
1	0.60	0.01
2	0.54	0.02
3	0.55	0.02
4	0.64	0.02
5	0.54	0.02
6	0.44	0.02

### Phenotypic correlations

**Fig. 4:**
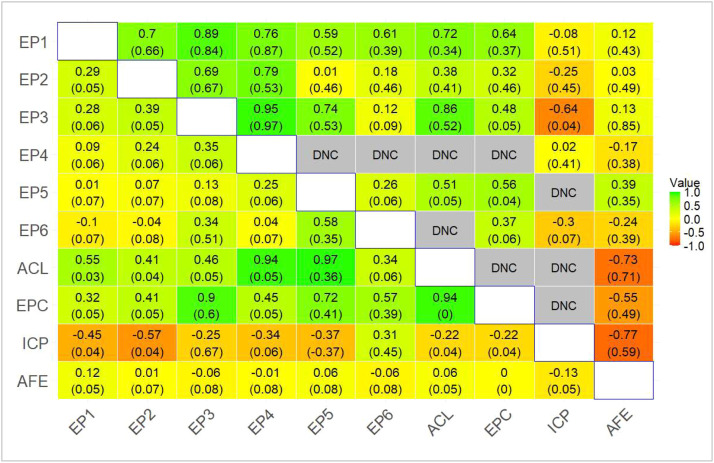
Estimates (standard errors) of genetic (upper triangle) and phenotypic (lower triangle) correlations among egg production periods. EP – egg production for period 1-6 EW – egg weight for periods 1-6 AC – average clutch length EPC – eggs per clutch ICP – inter clutch pause AFE – age at first egg.

Phenotypic correlation estimates among EP, clutch traits, and AFE are presented in Fig. 4. Only 81% of the bivariate analyses converged; those involving EP4 and ICP had the highest non-convergence rate due to limited data (Fig. 4). A larger sample size is expected to improve the computational efficiency and convergence. Phenotypic correlations among EP traits decreased as the time between the periods increased. A high (0.58) correlation was observed between EP5 and EP6, and moderate estimates were observed between EP3 and both EP6 and EP4. The high correlation between periods in temporal proximity may be due to common genetic and environmental factors (Sawalha et al., 2005).

Estimates of phenotypic correlations between EP and EW were generally weak, except for periods 4 (0.39±0.06) and 5 (−0.49±0.49) (Fig. 8). Correlation estimates were very low in the early periods and non-significant in period 1, but increased progressively, reaching their highest values by period 4. The estimate of −0.01 for period 1 in our study is lower than the −0.23 reported by Oleforuh-Okoleh (2011) for the same period in Nigerian ecotypes.

For AFE, negative phenotypic correlations were observed with EP3, EP4, EP6, and ICP, and a zero correlation with EPC, while weak positive estimates were observed with EP1, EP2, and EP5 (Fig. 4). EP1, 2, and 5 showed weak positive correlations with AFE, while EP3, 4, and 6 had negative $r_{p}$, none of which were significantly different from zero (*p* > 0.05). There was no clear pattern in the strength and direction of correlation estimates over time, contrasting with previous reports of moderate to strong positive phenotypic correlations between AFE and egg number in indigenous chickens (Miyumo et al., 2023; Yang et al., 2023). Our close-to-zero estimates indicate that AFE provides little predictive value for egg production across different laying periods in this population. Yang et al. (2023) defined egg production as the number of eggs laid from the first egg until 66 weeks of age. This means that hens with earlier AFE had a more extended laying period, suggesting a negative correlation between AFE and egg production. Miyumo et al. (2023) defined egg production as the number of eggs laid up to 12 weeks from the onset of lay. In our study, the length of each laying period was fixed at 90 days, starting from the AFE, precluding the expected negative correlation with AFE.

Phenotypic correlation estimates of ACL and EPC with EP traits ranged from 0.32 to 0.97 (Fig. 4). This implies that hens with longer clutches tend, as expected, to have more eggs per clutch, and such hens also tend to lay more eggs. The negative phenotypic correlation between ICP and EP in periods 1 through 5 implies that hens with short pauses between clutches tend to lay more eggs than those with extended rest periods. Estimates of phenotypic correlations among EW traits (EW1-EW6) varied widely but were positive, ranging from 0.01 to 0.5 (Table 8). A pattern of stronger correlations among traits that are temporally closer to each other and weaker correlations among those that are farther apart was observed. The strongest phenotypic correlations were between EW4 and EW5 (0.50) and between EW3 and EW4 (0.45). Weak correlations were found between EW1 and EW4-EW6, and between EW2 and EW6, with estimates of 0.01, 0.07, 0.03, and 0.08, respectively. High phenotypic correlations between traits suggest that changes in one period may predict changes in the other. Most pairs, however, yielded low estimates, suggesting weak associations and limited predictive power across distant time periods. Similarly, Lin et al. (1995) found positive moderate phenotypic correlations between egg weight at 210 days and 280 days and between 210 days and 300 days of age in laying ducks.

**Table 8:** Estimates of phenotypic correlations among egg weights between 90-day egg production periods, starting from the onset of lay.

Periods	Correlation	Standard error
1-2	0.35	0.02
1-3	0.15	0.02
1-4	0.01	0.04
1-5	0.07	0.03
1-6	0.03	0.04
2-3	0.36	0.03
2-4	0.22	0.04
2-5	0.19	0.03
2-6	0.08	0.04
3-4	0.45	0.03
3-5	0.32	0.04
3-6	0.03	0.23
4-5	0.50	0.02
4-6	0.20	0.04
5-6	0.29	0.03

EW1- egg weight in the first 90-day laying period, up to the sixth period EW6.

Estimates of phenotypic correlations between AFE and EW were generally weak, ranging from −0.21 to 0.24, with EW4 having the highest estimate, which was significantly different from zero at *p* < 0.05 (Table 9). The negative phenotypic correlation between AFE and EW1 (−0.04±0.13) was lower than the 0.28±0.04 reported by Miyumo et al. (2023) for Kenyan ICs for the same period. The large discrepancy between our results and previous findings may be attributed to differences in genetic background and environmental factors among the populations studied. These weak phenotypic correlations suggest that AFE has limited predictive power for egg weight.

**Table 9:** Estimates of genetic and phenotypic correlations and their standard errors for age at first egg with egg weight in different 90-day periods of lay, starting from the onset of lay.

Period	Genetic	Standard error	Phenotypic	Standard error
1	−0.04	0.07	−0.04	0.13
2	0.06	0.09	−0.21	0.12
3	0.03	0.10	−0.09	0.11
4	−0.12	0.11	0.24	0.10
4	−0.09	0.10	0.20	0.22
6	−0.10	0.12	0.02	0.26

For the analyses of $r_{p}$, between EW and LAY, the only models that converged were for periods 2 and 6, with estimates of 0.03 and 0, respectively (Table 10). The results suggest that egg weight is not dependent on a hen's high- or low-performing status, contrary to the known negative correlation between egg number and egg weight (Anene et al., 2023).

**Table 10:** Estimates of genetic and phenotypic correlations and their standard errors between egg weight and LAY by 90-day period starting from the age at first egg.

LAY	EW	Genetic	Standard error	Phenotypic	Standard error
LAY1	EW1	-	-	-	-
LAY2	EW2	−0.21	0.18	0.03	0.08
LAY3	EW3	-	-	-	-
LAY4	EW4	-	-	-	-
LAY5	EW5	-	-	-	-
LAY6	EW6	−0.07	0.14	0.00	0.15

Where the model did not converge, values are replaced by "-", EW – egg weight.

Estimates of phenotypic correlations among LAY traits are shown in Fig. 6. Analyses of period 1 with periods 4, 5, and 6 did not converge. Estimates of phenotypic correlations tended to decrease with increasing temporal distance between traits, from moderate for consecutive periods (0.33 to 0.43) to small negative values (up to −0.10) for distant periods; however, none were significantly different from 0 (*p* > 0.05).

### Genetic correlations

All genetic correlations among EP traits were positive (Fig. 4). None differed significantly from zero except the correlation between EP5 and EP6. Estimates among early EP traits (EP1-EP3) exceeded 0.69. However, correlations below 0.3 were observed between EP2 and EP5 and EP6, as well as between EP3 and EP6, reflecting a numerical decline in genetic correlation as the time between observed traits increased. Strong positive genetic correlations between traits in temporal proximity can be explained by pleiotropy, in which different traits are controlled by common genes (Stearns, 2010). It also means a highly correlated response in other traits resulting from selecting for one trait.

Negative genetic correlations were observed between egg weight and egg production traits in the early periods (1 to 3), approximately zero in period 4, and numerically high (0.91) in period 6. Still, none were significantly different from zero (Fig. 8). A negative genetic correlation between EP and EW has been known for decades; Arthur and Abplanalp (1975) reported an estimate of −0.42 in turkeys, while Blyth (1952) reported an estimate of −0.20 in White Leghorn, which agrees with recent findings by Firozjah et al. (2015). Our negative genetic correlation estimates between EP and EW in the early production periods (1-3) suggest that selecting for increased egg production early on could lead to a decrease in egg weight. However, estimates should be interpreted with caution due to the large standard errors. Nevertheless, selection strategies must carefully balance the negative impact on egg weight while aiming to improve egg production.

**Fig. 5:**
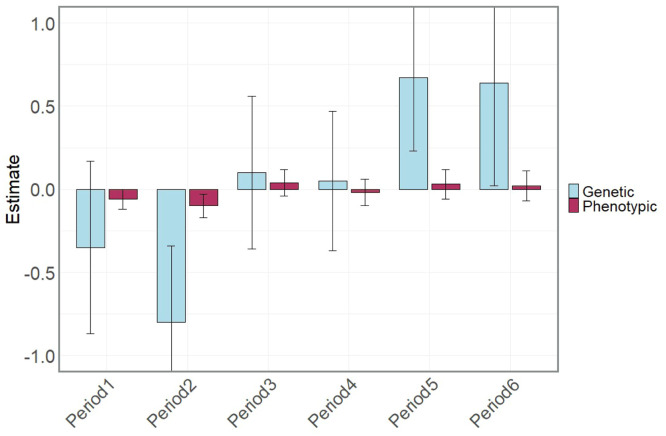
Estimates and standard error bars of genetic and phenotypic correlations between egg production and LAY by 90-day egg production period, starting at the onset of lay.

Genetic correlations between EP and LAY within the same period varied across periods, with estimates ranging from −0.80 to 0.67 (Fig. 5). Correlations were negative in periods 1 and 2, near zero in periods 3 and 4, and positive in periods 5 and 6. However, high standard errors indicated large uncertainties, and none were significantly different from zero (*p* > 0.05). No literature is available for direct comparison, especially for LAY traits, highlighting the need for further research into these genetic correlations.

Generally, weak positive genetic correlations were observed between EP and AFE. Exceptions were moderate positive estimates between EP5 and AFE, and weak negative correlations between AFE and both EP4 and EP6 (Fig. 4). However, none of the estimates were significantly different from zero (*p* > 0.05), suggesting that, if genetic factors exist, we lacked sufficient statistical power to detect them, despite the strong negative genetic correlation of −0.79 reported by Yang et al. (2023).

The EP traits were genetically positively correlated with ACL (0.38-0.86) and EPC (0.32-0.64) but negatively correlated with ICP (Fig. 4). However, the negative genetic correlations between ICP and EP2, EP3, and EP6 (−0.25, −0.64, and −0.30, respectively) indicate that genetic factors promoting higher egg production during these periods tend to reduce ICP.

Our results, however, showed estimates of genetic correlations between ACL and EP across periods ranging from 0.38±0.41 to 0.86±0.52, with correlations between ACL and EP1 and EP5 significantly different from 0 (*p* < 0.05), indicating that common genetic factors influence these traits. These estimates are consistent with those of Yang et al. (2023), who reported a high positive genetic correlation of ∼0.51 between egg number and ACL.

Our study revealed consistently high estimates of genetic correlations between EW across different periods, with values reaching up to 0.98 (Fig. 7), except for the correlation between EW1 and EW5, which was notably lower (0.27±0.14). A pattern of stronger correlations among traits that are closer to each other and weaker correlations among those that are farther apart was observed. Moderate correlations were found between EW5 and both EW1 and EW2. This suggests that changes in one period could be predictive of similar changes in adjacent periods. A distinct temporal pattern was observed for EW5, for which genetic correlations weakened as the time gap between EW5 and earlier periods increased. This temporal variation in genetic influence observed among trait pairs highlights the complexity of egg weight as a trait. It suggests that selection strategies targeting early egg weight may not be as effective for improving later egg weights. Most pairs, however, yielded low estimates, suggesting weak genetic associations and limited predictive power across distant time periods. Lin et al. (1995) found a strong positive $r_{g}$ (0.47 to 0.9) among EW traits at 210 days with 280 days and 300 days of age in laying ducks.

Estimates of genetic correlations between AFE and EW were generally negative and weak, ranging from −0.12 to 0.06 (Table 9). The genetic correlation between AFE and EW1 (−0.04) was far lower than 0.67 reported by Miyumo et al. (2023) for EW and AFE. Most estimates were not significantly different from zero, likely due to limited data.

**Fig. 6:**
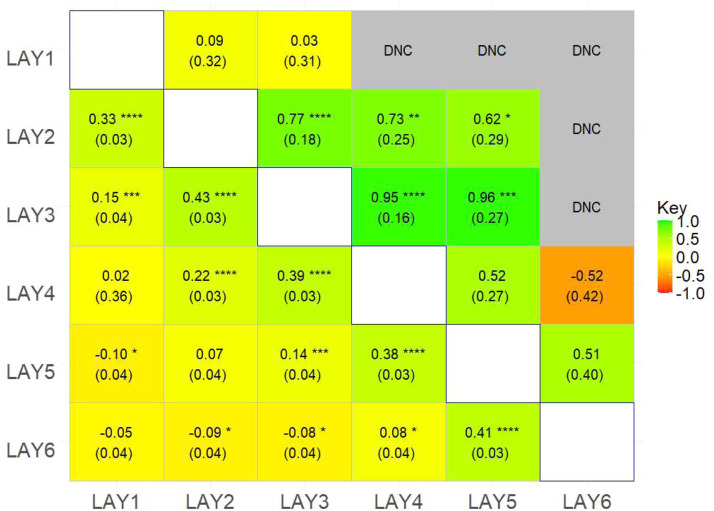
Estimates (standard errors) of genetic (lower triangle) and permanent environmental correlations (upper triangle) among LAY traits by 90-day period (1 to 6), starting from the age at first egg. DNC – the model did not converge Significance level: 0.05 - *, 0.01 – **, 0.001 ***, <0.001- ****.

For genetic correlations among LAY traits, a third of the models did not converge (Fig. 6). LAY in period 1 showed notably weak genetic correlations with LAY in the other periods. In contrast, LAY in period 4 had a negative correlation with LAY in period 6, which was not significantly different from 0. The remaining genetic correlation estimates were strongly positive and significantly different from zero (*p* < 0.05). Genetic correlations of LAY in period 3 with LAY in periods 2, 4, and 5 were highly significant (*p* < 0.05). Conversely, although the genetic correlation estimates for LAY between periods 5 and 6 were positive and high, they were not significantly different from 0. The estimates on the liability scale ranged from −0.27 to 0.64, compared with −0.24 to 0.39 on the observed scale (Table 6).

While phenotypic correlations can provide initial insights into trait relationships, they are unstable over time or across environments, as both environmental and genetic factors influence them. In contrast, genetic correlations tend to be more consistent. Genetic correlations and heritability estimates are more relevant for informing selection strategies, as they account for the underlying genetic basis of the trait and are not influenced by environmental factors.

### Permanent environmental correlations

**Fig. 7:**
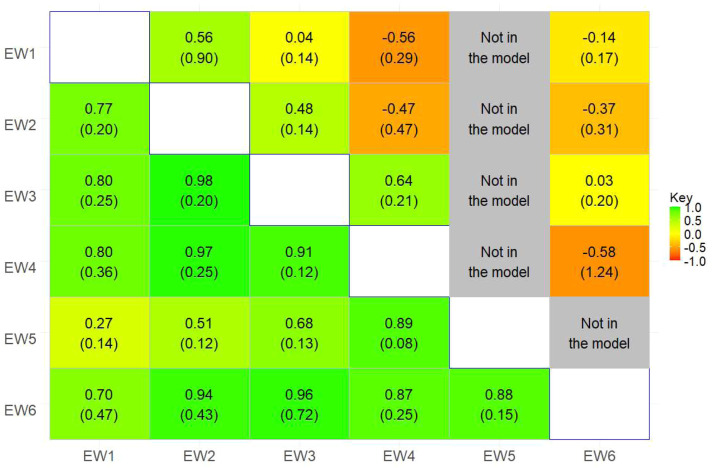
Estimates (standard errors) of genetic (lower triangle) and permanent environmental (upper triangle) correlations among egg weight in period 1-6. EW1 – egg weight in period 1.

Permanent environmental correlations ($r_{pe}$) among EW traits varied widely, from −0.58 to 0.64 (Fig. 7). The pe variance for EW5 converged to zero, and therefore, estimates of $r_{pe}$ involving EW5 were not available. Strong positive estimates were observed between EW3 and EW4, while moderate positive estimates were observed between EW1 and EW2. Additionally, EW2 and EW3 showed a moderate positive $r_{pe}$ correlation. In contrast, moderate negative $r_{pe}$correlations were observed between EW1 and EW4, as well as between EW2 with both EW4 and EW6. A moderate negative estimate was observed between EW2 and EW6, whereas the pe correlation between EW3 and EW6 was very low. The low pe correlations observed between EW6 and the early weights EW1, EW2, and EW3 suggest that long-term environmental factors are weakly related across multiple measurements for the same individual.

**Fig. 8:**
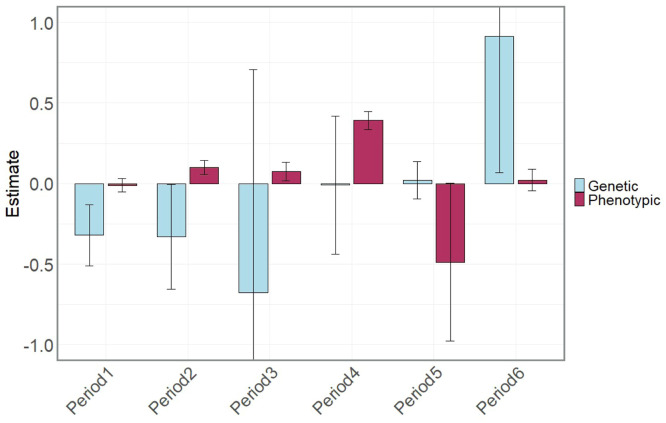
Estimates and standard error bars of genetic and phenotypic correlations between egg production and egg weight by 90-day egg production period, starting from the onset of lay.

## Conclusions

ICs are widely used in low-income countries due to their adaptability to low-input systems, but they perform poorly in these settings. We recorded egg production and egg quality data for Tanzanian ecotypes and estimated genetic parameters as a basis for genetic selection to improve performance.

ICs laid fewer and lighter eggs than commercial hens, and they took a long time to start laying, which increases production costs and delays the return on investment. ICs have very short clutch lengths and long interclutch pauses, consistent with natural brooding behavior.

Ecotypes differed in egg production, weight, and AFE; Kuchi performed best, and Ching'wekwe performed worst. This study revealed that IC ecotypes exhibit some level of genetic diversity, which influences egg production and egg weight. Specifically, ecotype differences and ancestral genetic composition both contributed to shaping egg production and weight traits.

We identified traits with a genetic basis and those without, providing targets for selective breeding. This study provides valuable insights for designing effective breeding strategies tailored to the unique characteristics of indigenous chickens.

## Declaration of AI and AI-assisted technologies in the writing process

Statement: During the preparation of this work, the authors used ChatGPT to improve readability. After using this tool, the authors reviewed and edited the content as needed and take full responsibility for the content of the publication.

## Funding

This study was made possible by the generous support of the American people through the United States Agency for International Development (USAID) Feed the Future Innovation Lab for Genomics to Improve Poultry (cooperative agreement number AID-OAA-A-13-00080).

## CRediT authorship contribution statement

**T. Magwaba:** Writing – review & editing, Writing – original draft, Visualization, Validation, Methodology, Investigation, Formal analysis, Conceptualization. **J.C.M. Dekkers:** Writing – review & editing, Supervision, Funding acquisition, Conceptualization. **S.J. Lamont:** Writing – review & editing, Funding acquisition, Conceptualization. **A. Wolc:** Writing – review & editing, Supervision, Methodology, Conceptualization. **E. Mollel:** Writing – review & editing, Data curation, Conceptualization. **J.R Mushi:** Writing – review & editing, Data curation, Conceptualization. **M. Walugembe:** Writing – review & editing. **E.N Amuzu-Aweh:** Writing – review & editing, Conceptualization. **G.H. Chiwanga:** Writing – review & editing. **N. Chouicha:** Writing – review & editing. **P.L. Msoffe:** Writing – review & editing, Conceptualization. **T. Kelly:** Writing – review & editing. **R.A. Gallardo:** Writing – review & editing. **A.P. Muhairwa:** Writing – review & editing, Conceptualization. **H. Zhou:** Writing – review & editing, Funding acquisition, Conceptualization.

## Disclosures

The authors declare that they have no financial or personal interests that could influence the results of this study.
